# Standardizing abortion research outcomes (STAR): a protocol for developing, disseminating and implementing a core outcome set for medical and surgical abortion^☆^

**DOI:** 10.1016/j.contraception.2016.12.009

**Published:** 2017-05

**Authors:** Katherine C. Whitehouse, Caron R. Kim, Bela Ganatra, James M.N. Duffy, Jennifer Blum, Dalia Brahmi, Mitchell D. Creinin, Teresa DePiñeres, Kristina Gemzell-Danielsson, Daniel Grossman, Beverly Winikoff, A. Metin Gülmezoglu

**Affiliations:** a The UNDP/UNFPA/UNICEF/WHO/World Bank Special programme of research, development and research training in human reproduction (HRP), World Health Organization, Department, Department of Reproductive Health and Research, Avenue Appia 20, 1211, Geneva 27, Switzerland; b Nuffield Department of Primary Care Health Sciences, University of Oxford, Oxford, OX2 6GG, United Kingdom; c Gynuity Health Projects, 15 East 26th Street, 8th Floor, New York, NY 10010, USA; d Ipas, PO Box 9990, Chapel Hill, NC 27515, USA; e Department of Obstetrics and Gynecology, University of California, Davis, 4860 Y Street, Suite 2500, Sacramento, CA, 95817, USA; f Fundación Oriéntame, Cra. 17 #33-50, Bogotá, Cundinamarca, Colombia; g Department of Women's and Children's Health, Karolinska Institutet and Karolinska University Hospital, 171 76 Stockholm, Sweden; h Department of Obstetrics, Gynecology and Reproductive Sciences, University of California, San Francisco, 1330 Broadway, Suite 1100, Oakland, CA 94612, USA

**Keywords:** CINAHL, Nursing and Allied Health Literature, COMET, Core Outcome Measures in Effectiveness Trials, COMIS, Core Outcome Measurement Instrument Selection, COS, Core Outcome Set, CROWN, Core Outcomes in Women's Health, GRADE, Grading of Recommendations Assessment, Development, and Evaluation, HIMC, Health Management Education Consortium, MARE, Medical Abortion Reporting of Efficacy, OMERACT, Outcome Measures in Rheumatology, PRISMA, Preferred Reporting Items for Systematic Reviews and Meta-Analyses, PROSPERO, Prospective Register of Systematic Reviews, STAR, Standardizing Abortion Research Outcomes, WHO, World Health Organization

## 1 Introduction

Approximately one in four of all pregnancies will end in induced abortion with an estimated 56 million taking place worldwide every year [1]. Medical and surgical abortions have low complication rates; however, due to the ubiquity of abortion and the potential for complications if performed unsafely or inappropriately, the need for robust clinical trials and sound guidelines and recommendations is clear [1], [2]. Besides the recent release of the Medical Abortion Reporting of Efficacy guidelines, there is a lack of standardized methods for selecting and reporting on outcomes in abortion clinical trials [3]. Variation amongst trials in outcomes and outcome measures limits the ability to compare, contrast and combine individual studies and draw meaningful conclusions. For example, the outcome of medical abortion success has been measured by some trials as the lack of receiving subsequent uterine evacuation and by others as follow-up endometrial stripe thickness on ultrasound. Researchers may contribute to outcome reporting bias by selecting outcomes based on factors like feasibility or reporting outcomes based primarily on statistical significance without assessing the clinical significance of any difference found. Oftentimes, the outcomes of clinical trials have been decided upon and collected with limited engagement from other stakeholders including women themselves. Outcomes such as stigma, fear of legal action, lack of information and harassment of medical staff are important aspects of the abortion process that are seldom included in clinical studies. Development and implementation of standardized outcomes and outcome measures in abortion clinical trials would improve the applicability of results and better guide research on what is one of the most common women's health practices [4].

Because heterogeneity in clinical trial outcomes can compromise the quality of systematic reviews and guidelines, a number of organizations have initiated efforts to standardize the process. The Core Outcome Measures in Effectiveness Trials (COMET) initiative was formed in 2010 to bring researchers, clinicians, patients and other interested persons or groups together to create a core outcome set (COS) for various medical research topics. A COS represents an agreed-upon minimum list of outcomes that should be measured and reported in any clinical trial on a certain subject [5]. “The existence or use of a core outcome set does not imply that outcomes in a particular trial should be restricted to those in the relevant core outcome set. Rather, there is an expectation that the core outcomes will be collected and reported, making it easier for the results of trials to be compared, contrasted and combined as appropriate; while researchers continue to explore other outcomes as well.” [6]. COMET has created a network of researchers developing COS and proposed a methodology for the process which includes systematic review of current outcomes, patient involvement, Delphi surveys and consensus establishment [5]. The Cochrane Collaboration has partnered with COMET to encourage the development, awareness and impact of COS on clinical research [4]. Information on COMET projects and COS is accessible to the public via a Web-based database (available online at: http://www.comet-initiative.org/).

In recognition of these research challenges and the importance of the COS, editors of over 70 women's health journals have come together to endorse the Core Outcomes in Women's Health (CROWN) initiative (available online at: http://www.crown-initiative.org/). CROWN aims to form a consortium of journals to promote COS, encourage researchers to develop COS using robust consensus methodology, promote the reporting of COS results, organize robust peer review and effective dissemination of manuscripts describing COS and facilitate the embedding of COS in research practice by working closely with researchers, reviewers, funders and guideline makers [7]. Currently, the development of COS is underway in many other fields of women's health including preeclampsia, preterm birth, endometriosis and postpartum hemorrhage [8], [9], [10]. Another example of a program that has been successful in addressing the issue of the COS is Outcome Measures in Rheumatology (OMERACT), which began an international initiative to improve OMERACT in 1992 in collaboration with the World Health Organization (WHO) [11]. Since then, OMERACT has successfully standardized trial endpoints focused on all aspects of rheumatology and implemented them in clinical and pharmaceutical trials to improve guidelines and reviews [11]. We aim to replicate the success of these projects to create a COS and corresponding core outcome measures for clinical trials on medical and surgical abortion.

### 1.1 Objective

We aim to produce, disseminate and implement a COS for medical and surgical abortion research.

## 2 Material & methods

Our COS on abortion has been prospectively registered with the COMET initiative under registration number 779 and is available online (http://www.comet-initiative.org/studies/details/779), and our systematic review is registered with International Prospective Register of Systematic Reviews under registration number CRD42016041876. Upon publication of this protocol, our COS will gain CROWN endorsement.

To technically support the project, we have formed an international advisory group with experience in abortion. The group includes abortion service providers, researchers, methodologists and client advocates from varied regions in both high and low or middle income countries. Within the advisory group, researchers at the WHO will serve as the study management team (WHO secretariat) to oversee daily activities of project tasks, with one member functioning as study coordinator. The study management team will be responsible for methodological development, recruitment of involved personnel, planning of processes and manuscript preparation. The management team will communicate with the rest of the advisory group either electronically or via telephone to discuss all critical stages of the project. Consensus will be used to make group decisions.

The Standardizing Abortion Research Outcomes (STAR) project will be divided into three stages; the first stage will identify possible core outcomes, the second stage will finalize the list of core outcomes and the third stage will define outcome measurements (i.e., definitions or instruments) for each selected outcome. Resulting findings will be widely distributed and promoted.

This work is supported internally by the WHO Department of Reproductive Health and Research, Development and Research Training in Human Reproduction program with no external funder.

### 2.1 Stage 1: identifying possible outcomes

#### 2.1.1 Systematic review

We will conduct a systematic review of randomized trials that evaluate induced abortion, both medical and surgical, following Preferred Reporting Items for Systematic Reviews and Meta-Analyses guidelines [12]. We plan to search PubMed, Embase, Cochrane Central Registrar of Controlled Trials and clinical trial registries. We will also hand-search reference lists to identify other possible studies. We will limit our search from 1980 onwards because medical abortion was not widely used until then [13]. We will include only studies published in any of the official United Nations languages (Arabic, Chinese, English, French, Russian and Spanish). We chose not to include less commonly spoken languages for which obtaining translation services would overly complicate the review process. We plan to include randomized controlled trials performed on women of all ages that evaluate medical and surgical abortion. We will exclude nonrandomized trials, animal studies, surveys, case series, case reports, cost–benefit analyses and observational studies. We will also exclude studies that report on spontaneous abortion (missed abortion, incomplete abortion) or fetal demise, pharmacokinetics or histology, animal trials and postabortion contraception.

Two reviewers will independently review all records obtained from database search and will assess relevant full-text articles. If there is disagreement about inclusion/exclusion of an article, the study management team will be involved to reach a consensus. Pertinent data such as type of abortion (medical vs. surgical), trimester, interventions studied, primary and secondary outcomes and outcome measures will be extracted from the included studies using a customized data extraction table and stored in a database. Clinical and woman-centered outcomes identified in this systematic review will be added to a list to be considered for entry into the modified Delphi method (see Section 2.2.1). Outcome measures extracted during this phase will be used later on during Stage 3 of the project.

#### 2.1.2 Qualitative review

An important, emerging component of COS methodology is the process of assessing and incorporating patient-centered outcomes [5]. Potential woman-centered outcomes for our project could include factors in decision-making, preferences, community knowledge/attitudes about abortion, information provided about abortion care and client–provider interactions. To ascertain these and other outcomes, we plan to perform a focused review of qualitative studies on the experiences of women and girls with medical and surgical abortion. We will search for relevant qualitative studies on PubMed, Embase, Cumulative Index to Nursing and Allied Health Literature, Cochrane Library, Global Health, PsycInfo, Google Scholar, Health Management Education Consortium, New York Academy of Medicine Gray Literature Report, Open Gray, Popline and Web of Science. We will also hand-search the reference lists of included studies. Search criteria will be developed with the help of a researcher skilled in qualitative methods, using purposive sampling techniques, to isolate key woman-centered outcomes reported in abortion literature. We plan to employ the same date restrictions as the aforementioned systematic review of clinical trials. We will include qualitative studies that explore women's experiences with induced medical and surgical abortion. We will exclude qualitative studies on women's experience with spontaneous abortion and fetal demise.

Two reviewers will independently read the full-text reports of all selected studies. They will extract data pertaining to woman-centered outcomes or themes such as satisfaction, perception of support by healthcare staff or abortion stigma, into a customized data extraction form and store in a database. Reviewers will identify key concepts and themes while continuously comparing these concepts and themes across all included studies throughout the review process. Similarities and conflicts in concepts and themes across studies will be identified and discussed amongst the qualitative reviewers and the study management team to derive a final list. Quality of studies will be assessed using an adapted version of the Critical Appraisal Skills Programme tool. These final themes and concepts will be considered as possible outcomes in the COS for clinical trials on abortion.

#### 2.1.3 Outcome inventory

Based on the information garnered from the systematic review and the qualitative review, we will create a comprehensive list of potential core outcomes to be used in the subsequent stages. The advisory group will be involved in this process and will be invited to suggest additional outcomes not otherwise included in the list.

### 2.2 Stage 2: determination & dissemination of core outcomes

#### 2.2.1 Modified Delphi method

Delphi is “an iterative multistage process designed to combine opinion into group consensus” [14]. The technique consists of sequential questionnaires which are distributed to and answered by participants [15]. In each stage, participants anonymously offer their opinion, and their input is fed back to subsequent stages of the survey process until consensus is reached. Modified Delphi methodology allows respondents to express their opinions and provide feedback to the input given by other participants in a noncoercive environment [15].

We plan to engage representatives of key abortion interest groups to participate in the surveys. Although there is no established minimum number of participants for Delphi, we aim to involve 200 respondents with an approximate even distribution of abortion service providers (physicians, midwives), researchers and women who have experienced medical or surgical abortions [15]. We will invite participants from diverse cultural, geographic and socioeconomic backgrounds. Abortion service providers will be recruited via appropriate professional societies and organizations. Board members of relevant peer-reviewed medical journals and professional societies will also be invited. Researchers will be invited to join if they have published studies included in our systematic and/or qualitative review. Women who have experienced abortion will be recruited via partnering abortion client representative or advocacy groups. Delphi surveys will be made available in written form for those without reliable Internet access.

Before administering the survey to participants, the study management team will perform a pilot test to verify ease of usability. After the pilot test has been successfully undertaken, we will begin recruiting possible respondents via email. Invitations will include a brief introduction to the STAR project, information about the Delphi process, estimated time commitment, importance of completing all stages and a link to the electronic survey. Each recruited participant will be given a unique username to access the Delphi survey online and will be asked to provide demographic and contact details and agree to participate in all rounds. We will offer monetary incentives to all abortion client participants who complete the entire Delphi process. Email reminders will be sent to participants to improve attrition rates. Those who do not respond to the initial email invitation will not be further contacted.

From the information gathered in Stage 1 of the STAR project, we will create a preliminary core outcomes list to be evaluated throughout the modified Delphi process. We plan to perform at least two rounds of Delphi surveys. In round one, we will present a list of outcomes in alphabetical order and ask participants to rank the importance of each outcome on a 9-point Likert scale from 1 to 9 wherein 1 signifies “not important” and 9 signifies “critical.” This scale is based on the Grading of Recommendations Assessment, Development, and Evaluation ranking system and is routinely used by other COS developers during the Delphi process [8]. Respondents will be invited to contribute additional outcomes to the list that they feel are important and not otherwise mentioned.

All outcomes from round one will be carried on to round two, including the additional outcomes suggested by respondents after the study management team reviews them. The list of original outcomes will be presented along with the percentage of participants scoring individual outcomes at each possible response, the tabulation for each individual stakeholder group (providers, researchers, women) and overall median and interquartile ranges from round one. In addition, participants will be able to view the specific score they assigned each outcome in the previous round. Participants will be asked to score the outcomes again, taking into account the feedback from round one. At the completion of round two, the study management team will calculate median and interquartile ranges and determine if consensus has been reached. “Consensus in” will be determined when at least 70% of participants scored an item as 7–9 (critical) and less than 15% score it as 1–3 (not important). “Consensus out” will be designated when at least 70% of participants score it 1–3 (not important) and less than 15% score it as 7–9 (critical). Outcomes that cannot be classified in either of these categories will be labeled as *no consensus* [8], [14]. Following analysis of the first two rounds, the study management team will determine whether or not an additional round of Delphi is warranted.

#### 2.2.2 Consensus meeting

After completion of the systematic review, qualitative review and modified Delphi method, we will organize a face-to-face meeting (including teleconference participation) between the advisory group and the study management team with the aim of reaching consensus and planning dissemination. If the Delphi process has been unsuccessful at reaching full consensus on all of the suggested outcomes, the group will discuss the remaining outcomes labeled *no consensus*. If the Delphi process did result in a clear consensus, then it will not be necessary to hold a consensus discussion at the meeting as this would undermine the extensive process already undertaken by the numerous participants in the Delphi surveys. In this case, the face-to-face meeting will still occur to primarily discuss dissemination plans. Once consensus has been sufficiently reached, the advisory group will perform a quality assessment to the COS during the face-to-face meeting. Quality assessment will follow the framework created by the OMERACT group, which involves evaluating each outcome for truth, discrimination and feasibility [16].

#### 2.2.3 Dissemination of COS

Discussions on the plans for dissemination of the COS will begin at the consensus meeting. Participants will be asked to consider implementing the COS in future relevant clinical trials and promoting COS use amongst colleagues and relevant professional societies. The study management team will prepare a manuscript detailing the methods and findings of the STAR project that will be sent to the advisory group for feedback before submitting to a peer-reviewed journal. We also plan to post our COS information on the COMET and CROWN Web sites as well as the WHO Reproductive Health Web site. The accepted manuscript will be disseminated to all Delphi survey participants, relevant professional societies, the Cochrane Collaborative and reproductive health medical journals. We anticipate presenting this project at scientific conferences and potentially scheduling additional meetings of abortion researchers at the national and international conferences to further promote dissemination.

### 2.3 Stage 3: determination of how abortion core outcomes are measured

After successfully determining what outcomes abortion trials should include, the next step is to ascertain how these outcomes should be measured. The process of creating core outcome measures is still an evolving methodology. The Core Outcome Measurement Instrument Selection (COMIS) project has suggested a method for determining outcome measures [17]. The process involves developing a list of current outcome measures in the literature based on a systematic review. We will extract these data during our aforementioned systematic review. Various methods for choosing outcome measurement instruments have been suggested, including Delphi surveys, expert panels and consensus meetings. We plan to follow the framework of the COMIS project as it continues to evolve and apply their process to the STAR project. At the completion of Stage 3, we aim to assign at least one high-quality outcome measurement to each outcome in the abortion COS.

## 3 Discussion

The STAR project will be the first COS in abortion, contributing to the field by generating a robust list of peer-reviewed, standardized outcomes and outcome measures. The impact of the STAR project will be multifaceted, serving to guide the development of future clinical trials, advise trial reporting in collaborating peer-reviewed journals, improve the strength of literature reviews and subsequent guideline creations and further advance and promote work in the field of COS development.

Although the STAR project will be promoted as a framework in the development of clinical trials in abortion research, the intent of the COS is not to limit researchers but, rather, provide them with a minimum list of outcomes to consider including in their projects or trials. The widespread adoption of these standardized abortion research outcomes will improve the applicability and comparability of the body of evidence in abortion research. Relevant medical journals and professional societies will be essential to the implementation of this work. Via the CROWN initiative, we anticipate garnering the support of a myriad of women's health journals [7]. As COSs become more common in the field of obstetrics and gynecology, we foresee that journals will begin requiring potential authors to either include peer-reviewed outcomes in their trials or comment on reasons for the deficiency of COS inclusion. Our work will help to promote the initiative to standardize clinical outcomes and add to the literature on COS development in women's health.

Abortion trials that include components of the COS in their study design will improve researchers' ability to draw meaningful conclusions from subsequent studies. Standardization in the reporting of outcomes will improve the results of systematic reviews and meta-analyses on abortion issues. Guidelines and recommendations based on these studies will be more robust, and thus potentially more likely to be adopted. When trials include core outcomes that were chosen based on input from clinicians, researchers and patients, resulting evidence may be more meaningful and applicable. Ultimately, we hope that the standardization in trial outcomes will lead to an improvement in the quality of evidence-based abortion care that can be delivered by the service provider to the woman.
